# Population pharmacokinetics and model-based dosing evaluation of bedaquiline in multidrug-resistant tuberculosis patients

**DOI:** 10.3389/fphar.2023.1022090

**Published:** 2023-03-27

**Authors:** Ge Shao, Ziwei Bao, Lina Davies Forsman, Jakob Paues, Jim Werngren, Katarina Niward, Thomas Schön, Judith Bruchfeld, Jan-Willem Alffenaar, Yi Hu

**Affiliations:** ^1^ Department of Epidemiology, School of Public Health and Key Laboratory of Public Health Safety, Fudan University, Shanghai, China; ^2^ The Fifth People’s Hospital of Suzhou, Infectious Disease Hospital Affiliated to Soochow University, Suzhou, China; ^3^ Department of Infectious Diseases, Karolinska University Hospital, Stockholm, Sweden; ^4^ Department of Medicine, Division of Infectious Diseases, Karolinska Institutet Solna, Stockholm, Sweden; ^5^ Department of Biomedical and Clinical Sciences, Linköping University, Linköping, Sweden; ^6^ Department of Infectious Diseases, Region Östergötland, Linköping University Hospital, Linköping, Sweden; ^7^ Department of Microbiology, Public Health Agency of Sweden, Stockholm, Sweden; ^8^ Department of Infectious Diseases, Kalmar County Hospital, Kalmar, Linköping University, Linköping, Sweden; ^9^ University of Sydney, Faculty of Medicine and Health, School of Pharmacy, Sydney, NSW, Australia; ^10^ Westmead Hospital, Sydney, NSW, Australia; ^11^ Sydney Institute for Infectious Diseases, University of Sydney, Sydney, NSW, Australia

**Keywords:** bedaquiline, multidrug-resistant tuberculosis, population pharmacokinetic modeling, dosage evaluation, target attainment

## Abstract

**Aims:** Bedaquiline is now recommended to all patients in the treatment of multidrug-resistant tuberculosis (MDR-TB) using standard dosing regimens. As the ability to measure blood drug concentrations is very limited, little is known about drug exposure and treatment outcome. Thus, this study aimed to model the population pharmacokinetics as well as to evaluate the currently recommended dosage.

**Methodology:** A bedaquiline population pharmacokinetic (PK) model was developed based on samples collected from the development cohort before and 1, 2, 3, 4, 5, 6, 8, 12, 18, and 24 h after drug intake on week 2 and week 4 of treatment. In a prospective validation cohort of patients with MDR-TB, treated with bedaquiline-containing standardized regimen, drug exposure was assessed using the developed population PK model and thresholds were identified by relating to 2-month and 6-month sputum culture conversion and final treatment outcome using classification and regression tree analysis. In an exploratory analysis by the probability of target attainment (PTA) analysis, we evaluated the recommended dosage at different MIC levels by Middlebrook 7H11 agar dilution (7H11).

**Results:** Bedaquiline pharmacokinetic data from 55 patients with MDR-TB were best described by a three-compartment model with dual zero-order input. Body weight was a covariate of the clearance and the central volume of distribution, albumin was a covariate of the clearance. In the validation cohort, we enrolled 159 patients with MDR-TB. The 7H11 MIC mode (range) of bedaquiline was 0.06 mg (0.008–0.25 mg/L). The study participants with AUC_0-24h_/MIC above 175.5 had a higher probability of culture conversion after 2-month treatment (adjusted relative risk, aRR:16.4; 95%CI: 5.3–50.4). Similarly, those with AUC_0-24h_/MIC above 118.2 had a higher probability of culture conversion after 6-month treatment (aRR:20.1; 95%CI: 2.9–139.4), and those with AUC_0-24h_/MIC above 74.6 had a higher probability of successful treatment outcome (aRR:9.7; 95%CI: 1.5–64.8). Based on the identified thresholds, simulations showed that the WHO recommended dosage (400 mg once daily for 14 days followed by 200 mg thrice weekly) resulted in PTA >90% for the majority of isolates (94%; MICs ≤0.125 mg/L).

**Conclusion:** We established a population PK model for bedaquiline in patients with MDR-TB in China. Based on the thresholds and MIC distribution derived in a clinical study, the recommended dosage of bedaquiline is sufficient for the treatment of MDR-TB.

## 1 Introduction

Multidrug-resistant tuberculosis (MDR-TB) is defined as tuberculosis (TB) where *Mycobacterium tuberculosis (Mtb)* is at least resistant to isoniazid and rifampicin. China is the world’s second-highest TB burden country, with 84,200 new TB cases and 17,528 MDR-TB cases in 2020 (World Health Organization, 2021). The treatment is long and associated with frequent adverse events, with a cure rate of MDR-TB as low as 59% in 2018 (Olaru et al., 2016; World Health Organization, 2021). Hence optimization of treatment is needed to achieve END TB goals.

Bedaquiline is a diarylquinoline drug approved by the U.S. Food and Drug Administration in 2012 and proved to significantly increase the success rate of MDR-TB treatment (Lounis et al., 2006; Diacon et al., 2012; Diacon et al., 2014; Healan et al., 2017; Olayanju et al., 2018; Li et al., 2019). Thus, bedaquiline is now recommended by WHO to all patients with MDR-TB, unless contraindicated (World Health Organization, 2019).

The drug concentrations and pharmacokinetics of anti-TB drugs vary widely among TB patients (Pasipanodya et al., 2013; McLeay et al., 2014). In the case of bedaquiline, drug exposure is related to body weight, albumin, age, race and concomitant use of rifampicin (McLeay et al., 2014; Van Heeswijk et al., 2014; Svensson et al., 2016; Svensson and Karlsson, 2017). The drug concentration of bedaquiline is important as activity has been found to be concentration-dependent, where the high concentrations of bedaquiline were associated with a faster decline in bacterial load in patients within 24 weeks (Tanneau et al., 2020) and sputum culture conversion after 6-month treatment (Zheng et al., 2021). Therefore, an optimal drug exposure of bedaquiline is important to improve the MDR-TB treatment.

Although there have been several studies to model the population pharmacokinetics (PK) of bedaquiline among healthy subjects and patients with drug-susceptible and multidrug-resistant TB (McLeay et al., 2014; Svensson et al., 2016), the threshold for drug exposure associated with optimal treatment outcome remains unknown. Therefore, the present study aimed to build a population PK model of bedaquiline among patients with MDR-TB, identify the threshold to predict the treatment in a prospective cohort of patients with MDR-TB as well as to evaluate the current doses by the probability of target attainment (PTA) analysis.

## 2 Methods

### 2.1 Study design and population

A multi-center prospective cohort study of patients with MDR-TB was conducted in Guizhou, Henan, Jiangsu and Sichuan Province in China between June 2016 to June 2019. The patients were aged between 18 and 70 years, diagnosed with MDR-TB by phenotypic drug susceptibility testing (DST), and had received a bedaquiline-containing regimen. Patients with abnormal liver or kidney function, pregnancy, or infected with human immunodeficiency virus, hepatitis B, or C virus were excluded.

From the prospective cohort study, we included subjects with rich sampling into a development cohort for pharmacological modeling, while those with limited sampling and a standardized WHO regimen were included into the validation cohort for identifying the threshold predictive of treatment outcome.

The participants in the development cohort were treated with a standardized bedaquiline-containing regimen for 6 months during the intensive phase, including bedaquiline, moxifloxacin or levofloxacin, linezolid as well as a background regimen to complete a full-oral regimen as we reported previously (Zheng et al., 2021), while those in validation cohorts received a standardized WHO regimen for 6 months containing bedaquiline, moxifloxacin, linezolid, clofazimine, and cycloserine. For both cohorts, the dosage of bedaquiline was 400 mg once daily for the first 2 weeks and 200 mg thrice weekly for the following 22 weeks, and the regimen for the following 18 months during the consolidation phase included at least 3 anti-TB drugs. Non-adherence was defined as discontinued for more than 3 days.

All study participants were routinely examined monthly during the intensive phase (the first 6 months) and once every 2 months during the consolidation phase (the next 18 months). A questionnaire was used to collect demographic data, while medical and laboratory data were extracted from hospital records.

**FIGURE 1:**
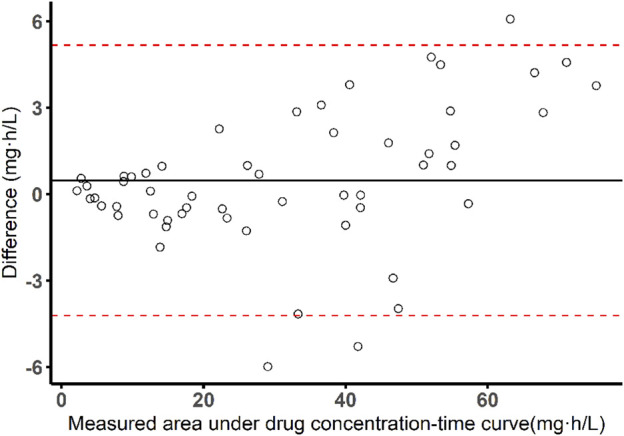
Bland-Altman plot of measured AUC *versus* predicted AUC values by Bayesian approach and limited sampling strategy (sampling time: 0, 2, 4, 6 h).

In the development cohort, blood plasma was sampled before and 1, 2, 3, 4, 5, 6, 8, 12, 18, and 24 h after drug intake on week 2 and week 4 of treatment respectively, while in the validation cohort, samples were collected predose and 2, 4 and 6 h after taking the drug on week 4 of treatment as limited sampling strategy indicated that the sparse concentrations performed well enough for simulation (Supplementary Table S1), and the consistency between the measured and predicted value of AUC_0-24h_ was evaluated by Bland-Altman analysis (Figure 1). After collection, the blood samples were centrifuged at 3,000 rpm for 10 min within 1 h, and the upper plasma was loaded into cryovials and stored at −80°C. Concentrations of bedaquiline were determined using high-performance liquid chromatography (HPLC) with a calibration curve ranging from 0.2 to 20 mg/L. The calibration curve was linear (*r* ^2^ = 0.9954) and accuracy and precision were between 91.3%–95.2%. The 5 samples of the development cohort were below the lower limit of quantification (10 ng/mL) and their assay results were ruled out from the analysis as a common way (Beal, 2001).

Sputum samples were collected at each visit and were sent to the prefectural TB reference laboratory for analysis. The bacterial cultures and MIC determination at baseline were performed using the Middlebrook 7H11 agar dilution method at bedaquiline concentration of 0.008–0.05 mg/L using serial two-fold dilution as previously described (Lounis et al., 2016). The Middlebrook 7H11 agar media (Becton Dickinson, Franklin Lakes, NJ, United States) were prepared according to CLSI document M07-A9 (Clinical and Laboratory Institute Standards, 2012). Quality control was performed under conditions equivalent to those of the experiment using *Mtb* strain H37Rv (ATCC 27294).

This study was carried out in accordance with the recommendations of the Declaration of Helsinki (2000). The protocol was approved by the ethics committee of the School of Public Health, Fudan University (IRB#2015-08-0565). Written informed consent was obtained from all participants.

### 2.2 Population pharmacokinetic modeling

Based on the development cohort, a population PK model was established using the non-linear mixed-effect method in Phoenix NLME (version 8.0; Certara Inc, Princeton, NJ, United States). Since bedaquiline has a unique dual zero-order input, we fixed the absorption rate as 1,000 h^-1^ based on a published population PK model (McLeay et al., 2014), resulting in the dose *via* the input compartment describing an initial zero-order (rather than first-order) input. The duration of infusion into the depot compartment (2.22 h), the duration of infusion into V_c_/F (1.48 h) and the fraction of dose into the depot compartment (58.5%) were also fixed from this reference for a better fit.

Inter-subject variability was estimated by exponential model for PK parameters as follows (Eq. 1):

$$\theta_{i}=\theta_{TV}\times \exp\left(\eta_{i}\right)$$

where θ_i_ is the parameter estimation for the *i*th individual, θ_TV_ is the typical value of the parameter estimation in the population, and η_i_ is the deviation from the population mean for the *i*th individual under the assumption that η_i_ are normally distributed with mean 0 and variance ω^2^. Furthermore, additive, proportional and combined-error models were explored to estimate the residual error variability.

The structural models were formulated through the objective function value (OFV), Akaike information criterion (AIC), and model goodness-of-fit plot. The models with the lowest values of OFV and AIC were regarded as superior. In the process of model development, weight, age, sex, height, and albumin were included as potential covariates to explain inter-individual pharmacokinetic variations.

The effects of continuous covariates such as weight, age, height and albumin were modeled using a power function after normalization by the population median (Eq. 2):

$$\theta_{i}=\theta_{TV}\times {\left(\frac{{cov}_{i}}{{cov}_{median}}\right)}^{\theta_{cov}}$$

Where cov_i_ is the covariate value for the *i*th individual, cov_median_ is the median value of the covariate. and θ_cov_ is the estimated parameter describing the fixed effect of covariates on the PK parameters.

For categorical covariates, such as sex, the effect was modeled as follow (Eq. 3):

$$\theta_{i}=\theta_{TV}\times \left(1+{cov}_{i}\times \theta_{cov}\right)$$

Where cov_i_ is a dummy variable that took on a value of 1 or 0.

The covariates selection was performed using stepwise regression with forward inclusion (△OFV>3.84, *p* < 0.05) and backward elimination (△OFV>6.64, *p* < 0.01). The covariates were selected or excluded depending on the value changes of OFV. The final model performance was authenticated by a visual predictive check (VPC). In the validation cohort, the pharmacokinetic parameters were calculated by Bayesian approach on the population PK model above.

### 2.3 Clinical-significant thresholds identification

The developed population PK model was applied for the validation cohort to calculate the pharmacokinetic parameters (C_max_, C_min_, AUC_0-24h_) on week 4 of treatment. Combined with the baseline 7H11 MICs of the clinical *Mtb* isolates, the ratios of bedaquiline drug exposure/susceptibility (C_max_/MIC, C_min_/MIC, AUC_0-24h_/MIC) were obtained. After that, the thresholds were identified by relating the drug exposure and ratio of drug exposure/susceptibility (C_max_, C_min_, AUC_0-24h_, C_max_/MIC, C_min_/MIC, AUC_0-24h_/MIC) to the 2-month sputum culture conversion as a marker of early treatment response, the 6-month culture conversion previously reported to be predictive of treatment outcome, and the successful treatment outcome, using the classification and regression tree (CART) analysis. The “rpart” package in R (version 4.1.2, R Foundation, Vienna, Austria) was used to generate the CART models with the default values specified in “rpart” (Atkinson and Therneau, 2000).

### 2.4 Dosage evaluation by probability of target attainment analysis

Based on CART-derived thresholds, the probability of target attainment was analyzed to evaluate the WHO recommended regimen (400 mg once daily for 14 days followed by 200 mg thrice weekly) (World Health Organization, 2020) as well as two suggested regimens (200 mg once daily, and 200 mg once daily for 56 days followed by 100 mg once daily) (Salinger et al., 2019) within a distribution of 7H11 MICs of 0.015, 0.03, 0.06, 0.125, 0.25, 0.5, 1 mg/L. Currently, 0.25 mg/L is suggested as a provisionary breakpoint for 7H11 by EUCAST (European Committee for Antimicrobial Susceptibility Testing, 2017). For each regimen, 1,000 patients were simulated based on the characteristics of the studied population. For the WHO recommended regimen and 200 mg once daily, we calculated the AUC_0-24h_ on week 4 of treatment, while the AUC_0-24h_ of 200 mg once daily for 56 days followed by 100 mg once daily was calculated on week 10 of treatment.

### 2.5 Statistical analysis

For demographic data, continuous variables were expressed as the interquartile range (IQR). The differences were compared using a t-test for normally distributed variables and the Mann-Whitney U test for non-normally distributed. Dichotomous variables were expressed as numbers and frequency (%). The differences were compared using the chi-square test. For drug exposure and susceptibility, pharmacokinetic parameters were expressed as median with interquartile range, MIC values were expressed as mode and range, and the Mann-Whitney U test was applied for comparison.

For validation of thresholds and clinical outcomes, a modified Poisson regression with robust standard errors was used to estimate the association between the CART-derived thresholds and the sputum conversion after 2 and 6 months’ treatment and successful treatment outcome. The time to sputum culture conversion was illustrated between patients above the CART-derived thresholds and those below using Kaplan–Meier curves and compared using the log rank test. The start point was the treatment initiation. The endpoint was defined as the beginning of consecutive two or more sputum culture conversions in the BACTEC MGIT960 system. The observation time was defined as the duration through start point until the endpoint. Censoring for study participants occurred when there was no sputum conversion observed throughout the whole MDR-TB treatment, death, or loss to follow-up. A Cox proportional hazards model was used to estimate the hazard ratio (HR). The association between thresholds and treatment outcome was adjusted by age, sex, place of residency, weight, adherence, and baseline time to liquid culture positivity as the possible confounders.

Data analyses and plotting were performed using R (version 4.1.2, R Foundation, Vienna, Austria). Levels of significance were set at 5% (α = 0.05), and a 95% confidence interval (CI) was calculated.

## 3 Results

### 3.1 Demographics and clinical characteristics

**FIGURE 2:**
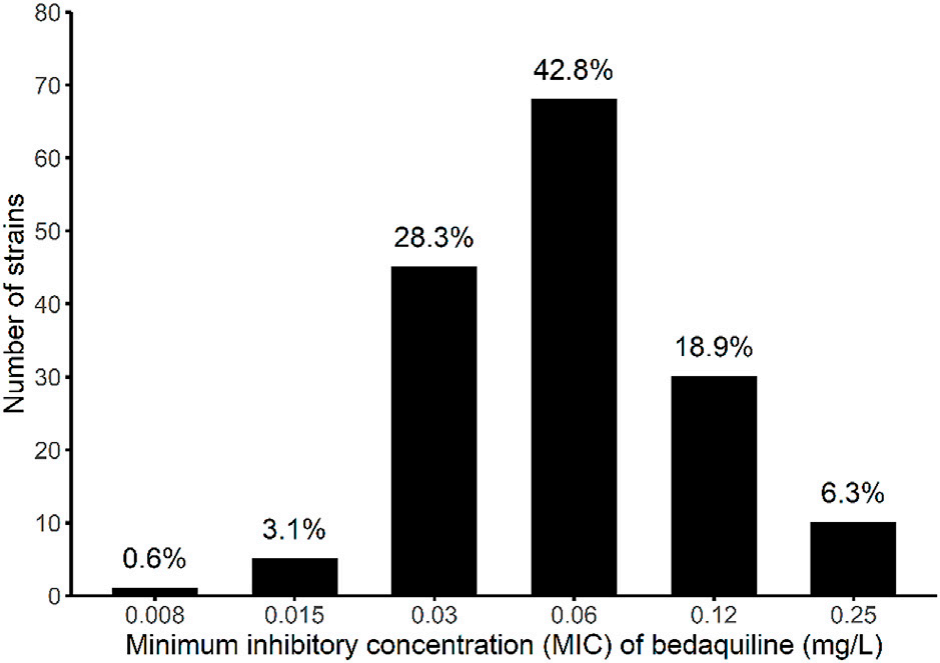
*Mycobacterium tuberculosis* minimum inhibitory concentration distribution of bedaquiline among the participants in the validation cohort.

Of the 55 study participants in the development cohort, 39 (70.9%) were male, and the median (IQR) age was 44 (34–53) years, while among the 159 patients in the validation cohort, 107 (67.3%) were male, and the median (IQR) age was 44 (29–54) years. The demographic and clinical characteristics of subjects were comparable between the two cohorts, except for weight and BMI (Table 1). The bedaquiline mode MIC (range) of the validation cohort, was 0.06 mg/L (0.008–0.25 mg/L). The majority of the MIC distribution was below 0.125 mg/L (93.7%; Figure 2). The bedaquiline MIC QC range for H37Rv was 0.015–0.06 mg/L, which was within QC range in the WHO report (World Health Organization, 2018).

**TABLE 1:** Baseline demographic and clinical characteristics of subjects.

Baseline characteristics	Development cohort (n = 55)	Validation cohort (n = 159)
Demographic data		
Age, years (median, IQR)	44 (34,53)	44 (29,54)
Sex (female) (n, %)	16 (29.1)	52 (32.7)
Weight, kg (median, IQR)	67 (59,74)	65 (59,71)
Height, cm (median, IQR)	165 (158,173)	166 (161,172)
BMI, kg/m2 (median, IQR)	23.8 (20.6,26.0)	24.6 (22.0,28.7)
Clinical data		
Albumin, g/L (median, IQR)	37.1 (32.0,40.6)	37.2 (32.4,41.6)
Diabetes Mellitus Type 2 (n, %)	9 (16.4)	27 (17.0)
Smoking (n, %)	6 (10.9)	31 (19.5)
Alcohol consumption (n, %)	5 (9.1)	28 (17.6)
Clinical severity (n, %)	11 (20.0)	41 (25.8)
Chest X-ray severity (n, %)	9 (16.4)	26 (16.4)
Pulmonary cavity (n, %)	9 (16.4)	33 (20.8)
TTP, h (median, IQR)	12 (11,14)	13 (11,14)
Pre -XDR^a^ (n, %)	9 (16.4)	13 (8.2)

BMI, body mass index; TTP, time to liquid culture positivity; Pre-XDR, pre-extensive drug resistance.

^a^ Pre-XDR-TB, TB, caused by *Mycobacterium tuberculosis* strains that fulfill the definition of multidrug-resistant and rifampicin-resistant TB, with additional resistance to any fluoroquinolone.

### 3.2 Population pharmacokinetic model

In the development cohort, five samples were below the lower limit of quantification (10 ng/mL) and ruled out of the analysis. Of the remained 1,205 bedaquiline concentrations from 55 subjects in the range of 0.04–5.96 mg/L were obtained for population PK modeling. Preliminary analysis of the base model showed the OFVs of the three- and four-compartment model with dual zero-order input (to capture dual peaks observed during absorption) were 763.6 and 758.0, respectively. Based on OFV, AIC, and diagnostic plots, the plasma concentrations of the development cohort were best described by a three-compartment model with dual zero-order input. A proportional error model was used to evaluate the residual variability. The pharmacokinetic parameters derived from the model included apparent clearance (CL/F) = 3.57 L/h and apparent distribution volume (V_c_/F) = 336.97 L (Table 2). Body weight and albumin were included in the final model. The body weight affected the clearance (CL) and the central volume of distribution (Vc), and the covariate effects of body weight on the central volume of distribution and clearance were fixed to 1 and 0.75, respectively, according to the principles of allometric scaling (Anderson and Holford, 2009). The albumin affected the clearance (estimate (CV%) = 3.76 (14.2)) (Table 2) (△OFV = −33.1, *p* < 0.01). Age, sex, and height had no significant effect on population PK parameters. The final model with the two covariates was as follows (Eq. 4, 5):

$$V_{c}/F\;\left(L\right)=336.97\times {\left(\frac{Weight}{67}\right)}^{1}\times \exp\left(\eta_{V}\right)$$

$$CL/F\;\left(L/h\right)=3.57\times {\left(\frac{Weight}{67}\right)}^{0.75}\times {\left(\frac{Albumin}{37.1}\right)}^{3.76}\times \exp\left(\eta_{CL}\right)$$

**TABLE 2:** Pharmacokinetics parameter estimations of the development cohort.

Parameter	Estimate (RSE%)	95% CI
Typical value parameter of population		
CL/F (L/h)	3.57 (11.9)	2.73–4.40
V_c_/F (L)	336.97 (12.6)	253.86–420.08
V_p1_/F (L)	2839.13 (46.3)	258.19–5420.06
CL_p1_/F (L/h)	2.97 (41.40)	0.56–5.39
V_p2_/F (L)	1391.89 (37.41)	370.16–2413.63
CL_p2_/F (L/h)	9.81 (18.86)	6.18–13.44
FR1 (%)	58.5	—
DUR1 (h)	2.22	—
DUR2 (h)	1.48	—
T_lag_ (h)	1.00 (9.1)	0.82–1.18
T_lag, add_ (h)	2.76 (21.0)	1.62–3.90
Covariable effect		
Weight effect on CL	0.75	—
Weight effect on V_c_	1	—
Albumin effect on CL	3.76 (14.2)	2.71–4.80
BSV		
ɷ^2^V	0.38 (67.99)	—
ɷ^2^CL	1.33 (166.76)	—
Residual variability		
σ^2^_add_	0.23 (2.75)	0.22–0.24

Abbreviations: RSE%, relative standard error; CI, confidence interval; F, apparent bioavailability; BSV, between subject variability; CL/F, apparent clearance; V_c_/F, apparent vol of distribution; V_p1_/F, apparent vol of distribution for the first peripheral compartment; CL_p1_/F, Apparent clearance between V_c_/F and V_p1_/F; V_p2_/F, apparent vol of distribution for the second peripheral compartment; CL_p2_/F, Apparent clearance between V_c_/F and V_p2_/F; FR1, fraction of dose into the depot compartment; DUR1, duration of infusion into the depot compartment; T_lag, add_, Additional lag time prior to administration of the remaining dose into Vc/F; DUR2, Duration of infusion into Vc/F; t_lag_, Lag time prior to absorption; The reference weight was 59 kg.

Where 336.97 L and 3.57 L/h were the typical value of V_c_/F and CL/F. The median weight was 67 kg. The median albumin was 37.1 g/L, and 3.76 indicates the relationship between CL/F and albumin.

**FIGURE 3:**
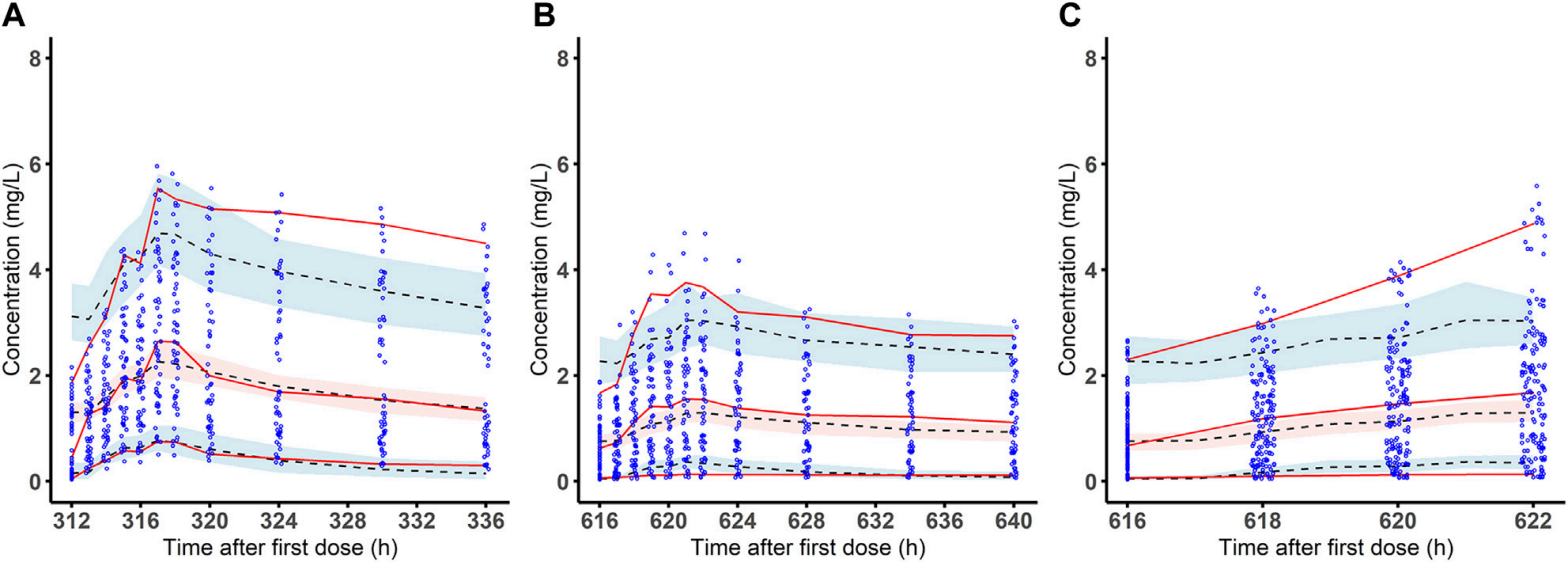
Visual predictive check of the final population pharmacokinetic model for bedaquiline. Observed bedaquiline concentration-time data and the visual predictive check (VPC) of the final model on week 2 **(A)** and week 4 **(B)** of treatment in the development cohort and on week 4 **(C)** of treatment in the validation cohort. The blue dots are the observed concentrations. The red solid lines are the 50th, 95th and fifth percentiles of the observed data. The dashed line in the middle and the dashed lines on the upper and lower sides are the 50th, 95th and fifth percentiles of the simulated concentrations, red and blue shaded areas are their model-predicted 95% CIs.

The VPC showed most of the observed values were distributed within the 95% CIs of predicted corresponding quantiles, which indicated the prediction of simulated data matched the observed values (Figure 3).

### 3.3 Thresholds identification

The validation cohort included 159 MDR-TB cases with 636 person-time concentration observations in the range of 0.16–5.28 mg/L. All of them completed 24 months’ MDR-TB treatment without death or loss of follow-up. During the treatment, 13 patients experienced bedaquiline discontinuation or dose reduction due to moderate and serious QT prolongation. As a result, 11 of 13 recovered and 10 resumed the standard dose. With the bedaquiline-containing regimen, 111 (69.8%) achieved sputum culture conversion after 2 months′ treatment, and 123 (77.4%) after 6-month treatment. During the treatment follow-up period, a total of 149 (93.7%) patients had sputum culture conversion with a median time (IQR) to conversion of 2 (1, 4) months. Among the 13 discontinued patients, 3 (23.0%) achieved sputum culture conversion after 2 months’ and 6 months’ treatment, and 8 (61.5%) at the end of treatment.

**FIGURE 4:**
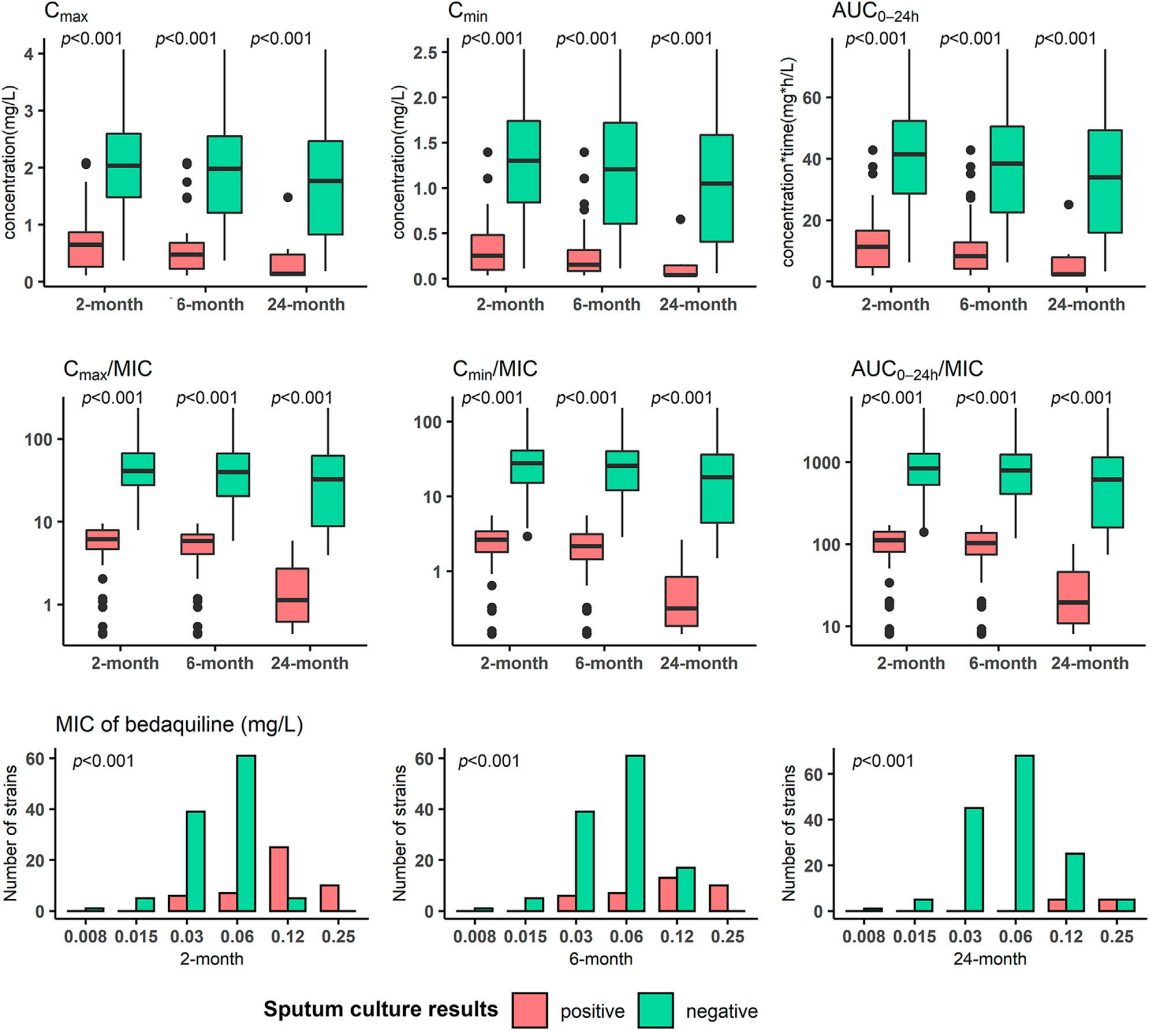
Distribution of bedaquiline exposure and susceptibility of *Mycobacterium tuberculosis* isolates respectively in participants with different treatment outcomes in the validation cohort. MIC: minimum inhibitory concentration; C_max_: maximum drug concentration; C_min_: minimum drug concentration; AUC_0-24h_: area under drug concentration-time curve over the last 24 h dosing interval. Mann-Whitney U test was applied for comparison.

In the validation cohort, the median (IQR) AUC_0-24h_ was 31.7 (13.9,48.5) mg⋅h/L, and the median (IQR) AUC_0-24h_/MIC was 557.8 (148.5,1059.9). A higher drug exposure on week 4 (C_max_, C_min_, AUC_0-24h_), especially for AUC_0-24h_, was observed among the patients with sputum conversion compared to those without, respectively, after 2 months’ (median AUC_0-24h_: 41.5 vs 11.4 mg⋅h/L), 6 months’ treatment (median AUC_0-24h_: 38.4 vs 8.3 mg⋅h/L), as well as with successful treatment outcome (median AUC_0-24h_: 34.0 vs 2.4 mg⋅h/L). The Middlebrook 7H11 MIC levels were also associated with sputum conversion at the observation time points. The difference was observed to be even more pronounced for AUC_0-24h_/MIC among patients with sputum conversion compared to those without after 2 months’ (median AUC_0-24h_/MIC:840.5 vs 112.3) and 6 months’ treatment (median AUC_0-24h_/MIC:795.9 vs 103.7), as well as for successful treatment outcome (median AUC_0-24h_/MIC:613.0 vs 19.6) (Table 3; Figure 4).

**TABLE 3:** Distribution of drug exposure and susceptibility among the participants with different treatment outcome in the validation cohort.

	2-month sputum culture			6-month sputum culture			24-month sputum culture		
	Positive(n = 48)	Negative(*n* = 111)	*P*	Positive (n = 36)	Negative (n = 123)	*P*	Positive (*n* = 10)	Negative (n = 149)	*P*
7H11 MIC, mg/L	0.12 (0.03,0.12)	0.06 (0.008,0.06)	<0.001	0.12 (0.03,0.25)	0.06 (0.008,0.06)	<0.001	0.12 (0.12,0.25)	0.06 (0.008,0.06)	<0.001
C_max_, mg/L	0.7 (0.26,0.87)	2.0 (1.48,2.60)	<0.001	0.5 (0.23,0.68)	2.0 (1.21,2.55)	<0.001	0.1 (0.12,0.48)	1.8 (0.83,2.46)	<0.001
C_min_, mg/L	0.3 (0.10,0.48)	1.3 (0.84,1.74)	<0.001	0.2 (0.08,0.32)	1.2 (0.61,1.72)	<0.001	0.04 (0.04,0.14)	1.1 (0.41,1.58)	<0.001
AUC_0-24h_, mg.h/L	11.4 (4.77,16.55)	41.5 (28.64,52.25)	<0.001	8.3 (4.20,12.76)	38.4 (22.52,50.49)	<0.001	2.4 (2.16,7.88)	34.0 (15.89,49.28)	<0.001
C_max_/MIC	6.2 (4.69,7.88)	41.1 (27.67,67.33)	<0.001	5.9 (4.07,7.03)	40.1 (20.47,66.82)	<0.001	1.1 (0.64,2.76)	32.7 (8.87,62.63)	<0.001
C_min_/MIC	2.6 (1.80,3.41)	27.9 (15.19,40.79)	<0.001	2.2 (1.45,3.14)	25.6 (12.10,40.23)	<0.001	0.3 (0.19,0.85)	18.1 (4.43,36.39)	<0.001
AUC_0-24h_/MIC	112.3 (80.72,141.52)	840.5 (527.64,1262.93)	<0.001	103.7 (74.38,137.33)	795.9 (409.49,1235.98)	<0.001	19.6 (11.32,46.46)	613.0 (160.42,1145.16)	<0.001

MIC: minimum inhibitory concentration; C_max_: maximum drug concentration; C_min_: minimum drug concentration; AUC_0-24h_: area under drug concentration-time curve. Pharmacokinetic parameters were expressed as median with interquartile range, MIC, values were expressed as mode and range, and the Mann-Whitney U test was applied for comparison.

**FIGURE 5:**
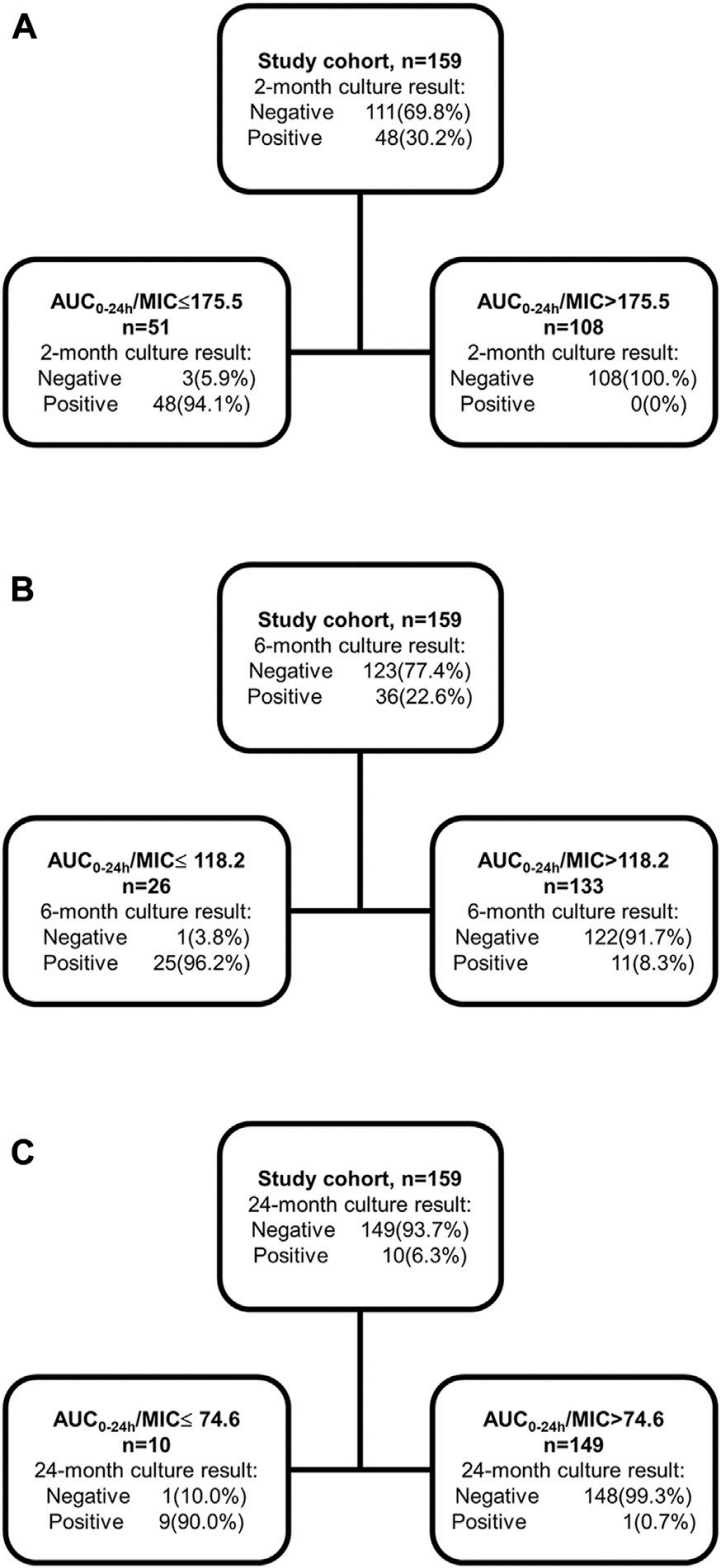
Classification and Regression Tree analysis for the thresholds to differentiate the 2-month, 6-month sputum conversion and successful treatment outcome in the validation cohort. Classification and Regression Tree analysis was applied to identify the thresholds predictive of 2-month **(A)**, 6-month **(B)** sputum conversion and successful treatment outcome **(C)**. AUC_0-24h_: area under drug concentration-time curve over the last 24-h dosing interval; MIC: minimum inhibitory concentration.

**FIGURE 6:**
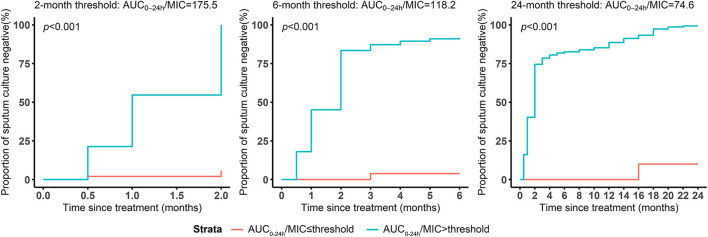
Survival analysis of months to sputum culture conversion grouped by the thresholds predictive of different treatment outcomes. The survival curves were illustrated to present the months to sputum conversion and compared using log rank test between the participants above and below the thresholds predictive of 2-/6- months sputum conversion and successful treatment outcome.

Regarding thresholds predictive of treatment outcome, the AUC_0-24h_/MIC of 175.5 was derived as the primary node to differentiate sputum culture conversion after 2 months’ treatment, with 118.2 for culture conversion after 6 months’ treatment and 74.6 for successful treatment outcome (Figure 5). Furthermore, the study participants with AUC_0-24h_/MIC above 175.5 had a higher probability of culture conversion after 2 months’ treatment compared with those below (adjusted relative risk, aRR:16.4; 95%CI: 5.3–50.4). Similarly, those with AUC_0-24h_/MIC above 118.2 had a higher probability of culture conversion after 6 months of treatment (aRR:20.1; 95%CI: 2.9–139.4), and those with AUC_0-24h_/MIC above 74.6 had a higher probability of successful treatment outcome (aRR:9.7; 95%CI: 1.5–64.8). This pattern was also seen for the association between the CART-derived thresholds and time to sputum conversion (Figure 6; Table 4).

**TABLE 4:** Association between the thresholds and sputum culture conversion among the participants in the validation cohort.

	Robust Poisson regression model			Cox proportional hazards regression model		
	Sputum culture conversion (%)	RR (95%CI)	aRR (95%CI)^a^	Median time (month) to culture conversion (IQR)	HR (95%CI)	aHR (95%CI)^a^
2-month threshold						
AUC_0-24h_/MIC≤175.5 (n = 51)	5.9	1	1	14 (5,19)	1	1
AUC_0-24h_/MIC>175.5 (n = 108)	100.0	17 (5.7–51)	16.4 (5.3–50.4)	1 (1,2)	27.3 (8.7–86.3)	29.4 (9.1–95.1)
6-month threshold						
AUC_0-24h_/MIC≤118.2 (n = 26)	3.8	1	1	18 (14,24)	1	1
AUC_0-24h_/MIC>118.2 (n = 133)	91.7	23.8 (3.5–163.1)	20.1 (2.9–139.4)	2 (1,2)	47.9 (6.6–344.9)	42.5 (5.9–308.9)
24-month threshold						
AUC_0-24h_/MIC≤74.6 (n = 10)	10	1	1	24 (24,24)	1	1
AUC_0-24h_/MIC>74.6 (n = 149)	99.3	9.9 (1.5–63.8)	9.7 (1.5–64.8)	2 (1,3)	36.4 (5–265.8)	31.3 (4.2–232.8)

RR: relative risk; CI: confidence interval; IQR: interquartile range; HR: hazard ratio.

^a^ Adjusted according to age, sex, place of residency, weight, adherence and baseline time to liquid culture positivity. Non-adherence was defined as discontinued for more than 3 days.

### 3.4 Exploratory analysis of current dosages in relation to Middlebrook 7H11 bedaquiline MICs

**FIGURE 7:**
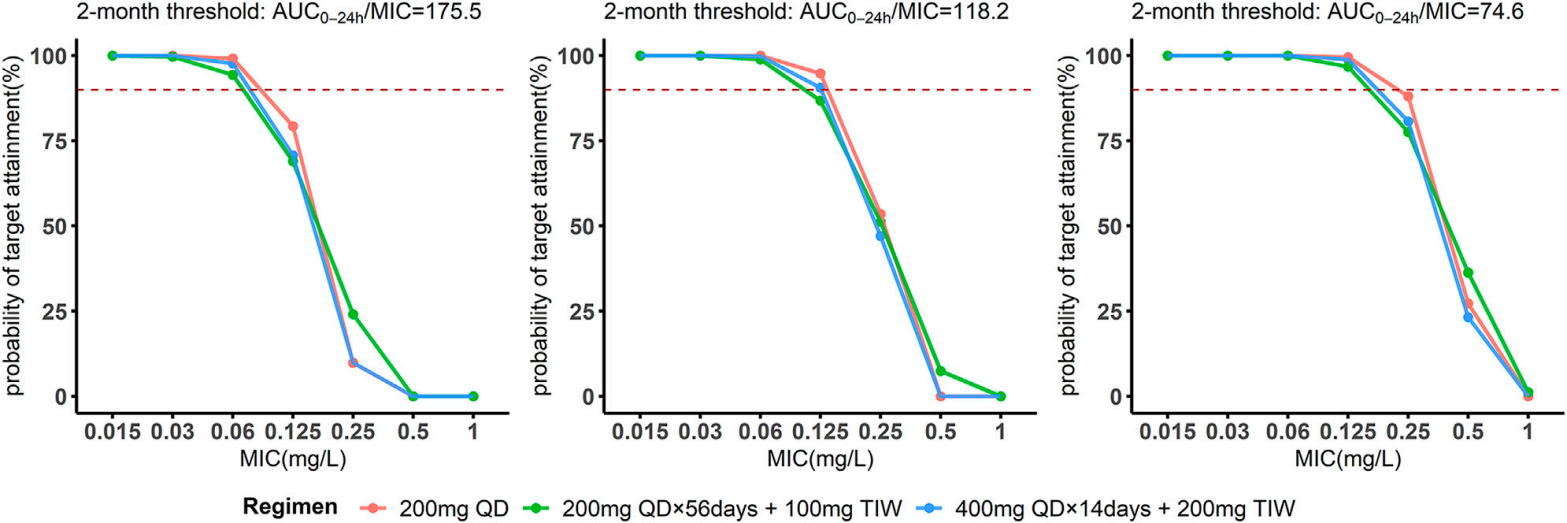
The probability of target attainment among the study participant from the validation cohort against varying 7H11 minimal inhibitory concentrations for bedaquiline. Probability of target attainment for bedaquiline taking the thresholds based on the 2-month, 6-month sputum conversion as well as successful treatment outcome as targets. The dashed lines indicate the 90% probability of target attainment.

Regarding the threshold related to sputum culture conversion after 2 months’ and 6 months’ treatment and successful treatment outcome, simulations showed that all regimens achieved a PTA of above 90% at MICs ≤0.06 mg/L. At an MIC of 0.125 mg/L, the WHO recommended dosage achieved 70.7% (2 months), 90.6% (6 months) and 98.9% (24 months) attainment based on the CART-thresholds related to different treatment outcomes, while 200 mg once daily was simulated to achieve 79.3% (2 months), 94.8% (6 months) and 99.6% (24 months) attainment. None of the studied regimens reached above 90% at MICs of 0.25 mg/L, 0.5 mg/L and 1 mg/L (Figure 7).

## 4 Discussion

In this study, we developed a bedaquiline population PK model in patients with MDR-TB and identified the thresholds related to the sputum conversion after 2 months’ (AUC_0-24h_/MIC = 175.5), 6 months’ treatment (AUC_0-24h_/MIC = 118.2) as well as successful treatment outcome (AUC_0-24h_/MIC = 74.6). Furthermore, these targets were obtained for the majority of the clinical isolates in our study.

Our results showed that the model to best describe the pharmacokinetic data was a three-compartment model with dual zero-order input, which is in agreement with a previously published study (McLeay et al., 2014), indicating that bedaquiline is extensively distributed in tissues and organs. The estimated mean CL value in our study of 3.57 L/h, was similar to the CL value of 2.78 L/h, in the aforementioned study (McLeay et al., 2014), and the V_c_/F of 336.97 L in the present study was comparably higher than published studies (McLeay et al., 2014; Svensson et al., 2016; Zhu et al., 2021). This may be due to the higher weight (median: 67 vs. 59 kg) of our subjects and using a standardized WHO regimen. In addition, the dual-zero input model was used to describe absorption, as the estimates of V_c_/F can be highly influenced by absorption. The covariates of the final model included body weight (affecting the CL and V_c_/F) and albumin (affecting the CL). The effect of albumin on PK disposition may be due to the high protein bounding of bedaquiline in plasma (Van Heeswijk et al., 2014).

We were able to validate the association between bedaquiline drug exposure/susceptibility and treatment outcome. The drug exposure on week 4 (AUC_0-24h_) was generally higher in subjects with sputum conversion after 2 months’ and 6 months’ treatment as well as subjects with the successful treatment outcome. The results support that the bactericidal activity of bedaquiline is concentration-dependent, as indicated by studies in a murine model of TB infection (Rouan et al., 2012). This may suggest that drug susceptibility is also a major factor influencing the treatment outcome, as previously reported (Liu et al., 2021). Furthermore, we found the ratio of drug exposure/susceptibility was most significantly associated with sputum culture conversion (Table 4). This confirms that the AUC_0-24h_/MIC is the main driver of the bactericidal effect (Van Heeswijk et al., 2014), also in well agreement with dose-fractionation experiments (Rouan et al., 2012). Additionally, by relating to the treatment outcome, AUC_0-24h_/MIC (175.5, 118.2, 74.6) was selected as the primary node to best differentiate the sputum culture conversion after 2 months’ and 6 months’ treatment as well as the successful treatment outcome. The dose simulation study of Salinger et al. indicates that the drug exposure remains stable through 4 weeks treatment until the end of treatment (Salinger et al., 2019). The pharmacokinetic data on week 4 is capable enough of estimating the drug exposure during the treatment with bedaquiline and thus the derived thresholds can be used as markers to predicate the short-term and long-term treatments (Kang and Lee, 2009).

In the present study, the current WHO dosage was observed to be effective for most study participants. Based on the identified thresholds predictive of 6 months’ sputum culture conversion and the successful treatment outcome, the WHO recommended regimen was able to achieve more than 90% target attainment at MICs ≤0.125 mg/L determined on Middlebrook 7H11 agar media. The vast majority of the *Mtb* isolates included in our study had MICs ≤0.125 mg/L (93.7%), which was similar to previous studies (Kaniga et al., 2016; Peretokina et al., 2020; Kaniga et al., 2022). However, similar to these studies, our MIC distribution was wide indicating poor reproducibility of individual MICs which was also observed in a systematic review by the WHO (World Health Organization, 2018). Additionally, it has been pointed out that it is very difficult if possible to separate technically from biological variability within the wild-type MIC distribution of any drug (Kahlmeter and Turnidge, 2022). Taking these caveats into account, our exploratory analysis showed that an MIC level close to the suggested breakpoint for Middlebrook 7H11 (≥0.25 mg/L), the recommended dosage may need further consideration. As 200 mg once daily has never been tested for longer than 8 weeks and that extensive metabolite accumulation is expected, this is not a current option without further studies. A previous study revealed that 200 mg once daily leads to a higher drug exposure level on week 24 of treatment than the WHO recommended regimen, while the highest metabolite M2 on week 24 of treatment does not exceed 600 ng/mL (Salinger et al., 2019). An open-label 8-week clinical trial found that 200 mg once daily showed efficacy comparable to the recommended regimen in the treatment of drug-susceptible tuberculosis, and there was no significant difference in the incidence of adverse events between the 200 mg once daily (3 out of 60 subjects had a QTc interval increase ≥60 ms) and the WHO recommended dosage (Tweed et al., 2019). In addition, a PK modeling study revealed that higher doses (the M2 concentration ≤1600 ng/mL) are not expected to lead to a substantially higher risk of QT prolongation (Tanneau et al., 2021). These observations highlight that therapeutic drug monitoring for bedaquiline would be beneficial in cases with high levels of MICs; however, a standardized drug susceptibility testing is urgently needed first, since the MIC of bedaquiline varies amply within and between methodologies.

One of the strengths of our study was that the population PK model was built based on a large number of sample points in real patients treated for MDR-TB, and the visual predictive check indicated that the model could be used for simulation purposes. Our study validated the concentration dependence of bedaquiline (Rouan et al., 2012), and further proposed the PK/PD thresholds relating to treatment outcomes after different lengths of treatment. The CART-derived threshold of 175.5 related to sputum conversion after 2-month treatment may be used to reach treatment efficacy for the short-treatment regimen, while the ones of 118.2 and 74.6 related to sputum conversion after 6 months’ treatment and successful treatment outcome respectively, may be used for dose adjustment for long-term treatment. In an exploratory analysis, we found that the current dosage was sufficient for most MDR-TB cases, and a dose increase might be required for *Mtb* isolates with resistance mechanisms to bedaquiline such as Rv0678 leading to MIC increases close to the epidemiological cut-off value (e.g., MIC ≥0.25 mg/L).

This study has several limitations. The model is limited by the lack of consideration of dynamic change during treatment in parameters such as albumin and weight, which have been observed to affect the population PK of bedaquiline (Svensson et al., 2016). Additionally, we did not include M2 in the analysis, since it was reported to be more likely to be the main driver for QT prolongation rather than the treatment efficacy (Janssen Pharmaceutical, 2012; Svensson et al., 2016). Due to the lack of sufficiently and clinically-validated targets for bedaquiline, the thresholds for efficacy were based on the principle that the recommended dosing regimen is appropriate from a safety and efficacy perspective. There is a need to further investigate the efficacy in the clinical trials to validate the targets. Additionally, as the study participants were included in the analysis if they were alive and had completed MDR-TB treatment throughout the whole course, this may restrict the representativeness of the study findings to some degree. Meanwhile, given the possible confounding from baseline disease status and adherence, the association between drug exposure and treatment outcome was adjusted for age, sex, area and adherence. Moreover, the PTA of studied regimens is calculated based on the threshold from drug exposure on week 2 or week 10 of treatment, which may not fully characterize the exposure throughout treatment. It also needs to be considered that the variability for MIC testing is substantial and affects target attainment. The results in this study are only valid for Middlebrook 7H11, which is not suitable for clinical routine due to a slow turn over time and complexity. Additionally, we did not account for the interaction from the concomitant use of other TB drugs, which may affect the pharmacokinetics of bedaquiline, like clofazimine (Hartkoorn et al., 2014; Hu et al., 2016; Xu et al., 2017; Liu et al., 2021).

## 5 Conclusion

We established a population PK model for bedaquiline in patients with MDR-TB in China. Based on the thresholds derived in a clinical study, the WHO recommended dose (400 mg once daily for 14 days followed by 200 mg thrice weekly) of bedaquiline is sufficient for the treatment of the majority of MDR-TB isolates.

## Data Availability

The original contributions presented in the study are included in the article, further inquiries can be directed to the corresponding author.
