# Supplementary material for: Population pharmacokinetics and model-based dosing evaluation of bedaquiline in multidrug-resistant tuberculosis patients
Source: Front Pharmacol. 2023 Mar 27;14:1022090. doi: 10.3389/fphar.2023.1022090 (PMC10083270; doi:10.3389/fphar.2023.1022090)
Supplement: Supplementary file 1 [file DataSheet1.docx]

Supplementary Material

**Supplementary Table S1. Limited sampling strategy of bedaquiline using Bayesian method and multiple linear regression in development cohort**

| **Sampling time (h)** | **Bayesian method** | | |  | **Multiple linear regression** | | | |
| --- | --- | --- | --- | --- | --- | --- | --- | --- |
|  | **MPE%** | **RMSE%** | **R^2^** |  | **AUC_0-24h,est_ calculation** | **MPE%** | **RMSE%** | **R^2^** |
| 0 | 24.9 | 28.5 | 0.936 |  | AUC_0-24h_=3.2+39.7×C0 | 20.7 | 18.9 | 0.917 |
| 4 | 11.3 | 12.4 | 0.965 |  | AUC_0-24h_=2.7+20.1×C4 | 13 | 11.9 | 0.967 |
| 6 | 12.4 | 13.2 | 0.963 |  | AUC_0-24h_=2.0+18.1×C6 | 9.2 | 9.3 | 0.98 |
| 0,4 | 9.2 | 11.7 | 0.988 |  | AUC_0-24h_=2.0+13.1×C0+14.1×C4 | 9 | 9.2 | 0.98 |
| 0,6 | 8.5 | 11.2 | 0.988 |  | AUC_0-24h_=1.6+10.1×C0+13.9×C6 | 6.3 | 7.5 | 0.987 |
| 2,6 | 11.8 | 11.7 | 0.972 |  | AUC_0-24h_=2.0+0.2×C2+17.9×C6 | 9.2 | 9.3 | 0.98 |
| 0,2,6 | 6.4 | 8.4 | 0.988 |  | AUC_0-24h_=1.6+12.2×C0+6.9×C2+8.0×C6 | 6.6 | 7.1 | 0.988 |
| 0,4,6 | 5.7 | 8.4 | 0.991 |  | AUC_0-24h_=1.5+10.0×C0+-2.5×C4+16.2×C6 | 6 | 7.5 | 0.987 |
| 2,4,6 | 11.1 | 10.5 | 0.978 |  | AUC_0-24h_=1.9+1.9×C2+-5.0×C4+21.1×C6 | 8.9 | 9.2 | 0.98 |
| **0,2,4,6** | **5.9** | **7.8** | **0.988** |  | **AUC_0-24h_=1.4+12.9×C0+10.2×C2+-8.4×C4+12.7×C6** | **5.9** | **6.7** | **0.99** |

AUC_0-24h,est_ means the estimated value of area under the concentration-time curve from 0 to 24h at week 4 based on the equation.

C0, C2, C3, C4, C5 and C6 are the blood concentration of bedaquiline at Predose and 2h, 3h, 4h, 5h and 6h after drug intake.

**Table S2. Population characteristics and pharmacokinetic data of the three regimens for the simulated population**

|  | 400mg QD for 14 days followed by 200mg TIW | 200mg QD | 200mg QD for 56 days followed by 100mg QD |
| --- | --- | --- | --- |
| Number of patients | 1000 | 1000 | 1000 |
| Pre -XDR (%) | 16.4 | 16.4 | 16.4 |
| AUC, mg·h/L (median, IQR) | 28.5 (20.6, 36.9) | 30.7 (23.3, 38.0) | 30.3 (19.6, 43.6) |
| Cmax, mg/L (median, IQR) | 1.4 (1.0,1.7) | 1.5 (1.2,1.8) | 1.4 (0.9,1.9) |

Pre-XDR, pre-extensive drug resistance; IQR, interquartile range; QD, once daily; TIW, three times weekly
